# Asset Transfers among those Accessing the Medicaid Program: Are Wealthy Older Adults Gaming the System?

**DOI:** 10.1093/geroni/igab046.3077

**Published:** 2021-12-17

**Authors:** Jane Tavares, Marc Cohen

**Affiliations:** 1 LeadingAge LTSS Center @UMass Boston, Boston, Massachusetts, United States; 2 University of Massachusetts Boston, University of Massachusetts Boston, Massachusetts, United States

## Abstract

Medicaid is the largest payer of long-term services and supports and millions of older Americans rely on the means-tested program for health care coverage. There has been longstanding concern that wealthy older adults are taking advantage of the program by divesting assets in order to qualify for coverage. The existing research on the issue is somewhat dated, does not focus on the question of asset transfer, and often lacks a significant longitudinal view. Thus, questions remain about whether states need to tighten asset eligibility rules to prevent the wealthier older adults from accessing the program. This analysis explores longitudinal data from the Health and Retirement Study (1998 to 2016) to determine the extent to which wealthier Americans age 50 and older engage in asset transfer to access Medicaid. Our findings demonstrate that this may occur among a relatively small proportion of wealthy older adults, and that tightening Medicaid eligibility criteria would likely have a small to modest impact on the financial status of the program.

